# Single T Cell Sequencing Demonstrates the Functional Role of *αβ* TCR Pairing in Cell Lineage and Antigen Specificity

**DOI:** 10.3389/fimmu.2019.01516

**Published:** 2019-07-31

**Authors:** Jason A. Carter, Jonathan B. Preall, Kristina Grigaityte, Stephen J. Goldfless, Eric Jeffery, Adrian W. Briggs, Francois Vigneault, Gurinder S. Atwal

**Affiliations:** ^1^Department of Applied Mathematics and Statistics, Stony Brook University, Stony Brook, NY, United States; ^2^Cold Spring Harbor Laboratory, Cold Spring Harbor, NY, United States; ^3^Watson School of Biological Sciences, Cold Spring Harbor Laboratory, Cold Spring Harbor, NY, United States; ^4^Juno Therapeutics, Seattle, WA, United States

**Keywords:** TCR–T cell receptor, CD4 and CD8 T cell repertoires, TCR repertoire diversity, single-cell sequencing, machine learning

## Abstract

Although structural studies of individual T cell receptors (TCRs) have revealed important roles for both the α and β chain in directing MHC and antigen recognition, repertoire-level immunogenomic analyses have historically examined the β chain alone. To determine the amount of useful information about TCR repertoire function encoded within αβ pairings, we analyzed paired TCR sequences from nearly 100,000 unique CD4^+^ and CD8^+^ T cells captured using two different high-throughput, single-cell sequencing approaches. Our results demonstrate little overlap in the healthy CD4^+^ and CD8^+^ repertoires, with shared TCR sequences possessing significantly shorter CDR3 sequences corresponding to higher generation probabilities. We further utilized tools from information theory and machine learning to show that while α and β chains are only weakly associated with lineage, αβ pairings appear to synergistically drive TCR-MHC interactions. Vαβ gene pairings were found to be the TCR feature most informative of T cell lineage, supporting the existence of germline-encoded paired αβ TCR-MHC interaction motifs. Finally, annotating our TCR pairs using a database of sequences with known antigen specificities, we demonstrate that approximately a third of the T cells possess α and β chains that each recognize different known antigens, suggesting that αβ pairing is critical for the accurate inference of repertoire functionality. Together, these findings provide biological insight into the functional implications of αβ pairing and highlight the utility of single-cell sequencing in immunogenomics.

## Introduction

With potentially up to 10^15^ unique αβ T cell receptor (TCR) pairs, a wealth of clinically-relevant information pertaining to infectious disease, autoimmunity, and cancer immunotherapy is encoded within the remarkable diversity of the TCR repertoire (1–3). As limitations in technology have historically precluded meaningful single-cell sequencing experiments, our current understanding of the TCR repertoires' diversity, structure, and function is almost entirely based on bulk-sequencing of the β chain repertoire alone (4–6). While such approaches have yielded impressive insights into adaptive immunity, they, *de facto*, are forced to make use of the assumption that the pairing of αβ TCR chains contains little useful information. In contrast, structural insights gleaned from a relatively small number of TCR-peptide-MHC structures have clearly defined important roles for both the α and β TCR chains in driving alloreactivity and antigen specificity (7–10). While our understanding of the underlying biology suggests that αβ pairings may themselves contain useful information on TCR function and repertoire diversity, whether this theoretical information can be approximated from bulk-sequencing, and if not, whether it can be utilized to meaningfully improve our understanding of the TCR repertoire remains largely a matter of conjecture.

While previous methods for paired αβ TCR sequencing have been developed (11–15), only recently have technological advances enabled high-throughput capture of paired αβ TCR sequences (16–18). We recently took advantage of one such single-cell sequencing method to capture more than 200,000 paired αβ TCR sequences from the peripheral blood of five healthy individuals, finding that the use of bulk and single-cell sequencing often resulted in significantly different diversity estimates (19). In the present study, we asked whether we could infer additional information about TCR repertoire function when examining paired αβ sequences relative to either of the single chain repertoires. Toward this, we used 10× Genomics single-cell platform (17) to add ~11,000 new αβ paired sequences to the ~86,000 CD4^+^ and CD8^+^ TCR sequences we previously obtained using the AbVitro method (18, 19). In addition to providing the most comprehensive comparison of the human CD4^+^ and CD8^+^ αβ TCR repertoires to date, we examined the ability of αβ pairings to provide information about T cell lineage and antigen specificity beyond that contained in the single-chain repertoires. At similar repertoire depths, we find that the paired αβ repertoire contains useful information about TCR function, both in terms of MHC recognition and antigen specificity, that is not accessible through conventional bulk-sequencing. Consequently, our study demonstrates the utility of using new single-cell sequencing approaches, in addition to conventional high-throughput bulk-sequencing, to capture a more accurate picture of TCR repertoire function.

## Results

###  Overlap Between the CD4^+^ and CD8^+^ Repertoires

During thymic positive selection, bipotent T cell precursors differentiate into either the CD4^+^ helper T cell or the CD8^+^ cytotoxic T cell lineage. Although this lineage selection process is contingent upon the interaction of the heterodimeric αβ TCR with either MHC class II or class I, respectively, understanding the general TCR features that mediate the TCR-pMHC interaction remains an area of active interest (20, 21). Potentially, the required ability to recognize structurally diverging MHC classes creates systematic differences in the CD4^+^ and CD8^+^ TCR repertoires. In support of this idea, previous studies have identified certain germline regions and CDR3 features in the single chain repertoires that are associated with up to ~5 times increase in likelihood for either CD4^+^ or CD8^+^ status (22–24). If αβ TCR pairing is an important component for understanding the differences between two TCR repertoires, we hypothesized that αβ pairs should be much less commonly shared between the CD4^+^ or CD8^+^ populations. That is, the information about αβ pairing should correlate with increased functional specificity for one of the two MHC classes.

**Figure 1:**
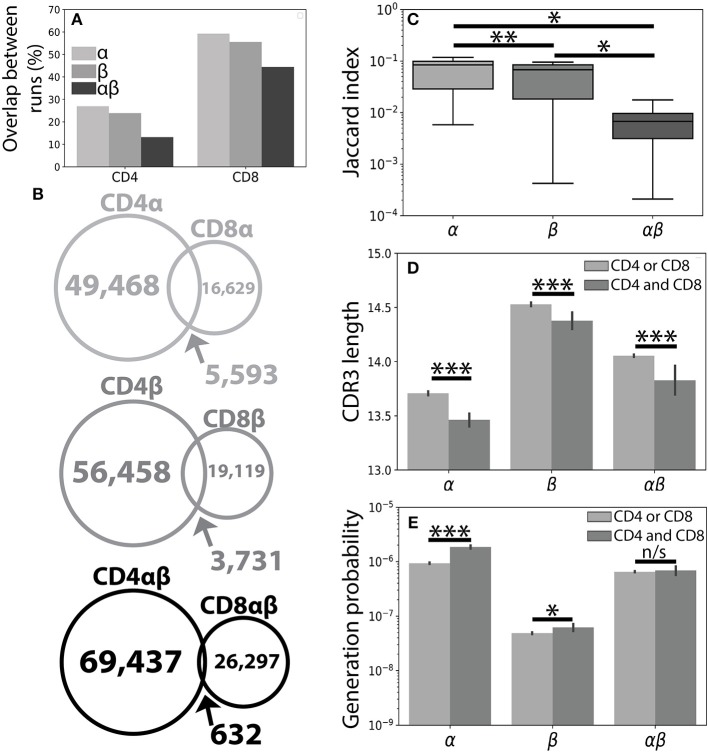
The CD4^+^ and CD8^+^ TCR repertoires are largely distinct. **(A)** A single peripheral sample from Subject 1 was sequenced using both the Abvitro (18) and 10× Genomics (17) platforms. The number of TCR clones found using both methods is reported as a fraction of the total number of TCRs observed in the considerably smaller 10× run. **(B)** Population unique TCRs, defined as Vαβ-CDR3αβ amino acid clonotypes, were calculated by combining CD4^+^ and CD8^+^ sequences from 7 healthy individuals. The overlap between CD4^+^ and CD8^+^ repertoires were calculated for the α (top), β (middle), and paired αβ (bottom) repertoires. **(C)** The Jaccard index quantitatively measures the overlap between the CD4^+^ and CD8^+^ repertoires separately for each individual. Significance between samples was assessed using a paired sample Student's *t*-test. **(D)** Bar plots show mean CDR3 lengths for α, β, and αβ TCR sequences found exclusively in (light gray) or shared between (dark gray) the CD4^+^ and CD8^+^ lineages. Error bars represent bootstrapped 99% confidence intervals of the mean with significance assessed using the Mann-Whitney *U* test. **(E)** The probability of generating a given CDR3 sequence was estimated using OLGA (25). αβ probabilities were calculated by assuming complete independence between the α and β chain. Shared sequences were more likely to be randomly generated than those found in only one of the repertoires. For all panels, n/s-not significant, ^*^p < 0.05, ^**^p < 0.01, and ^***^p < 0.001.

With this goal in mind, we first addressed how the paired αβ TCR repertoires differ between the CD4^+^ and CD8^+^ T cell populations, independent from an individual's HLA type (Supplemental Table 1). Toward this, we obtained paired αβ TCR sequences delineated by CD4^+^ and CD8^+^ lineage from our recently published work (19). In addition to these sequences captured using the AbVitro microfluidic platform (18), we resequenced samples from two individuals using the independent 10× Genomics single-cell sequencing platform (17). While we obtained only a small number of TCR sequences during resequencing, potentially due to RNA degradation secondary to prolonged storage times, a large fraction of these new TCR sequences were also found in the original dataset (Figure 1A). These findings strongly suggest the ability of both of these methods to accurately obtain TCR sequences in a high-throughput fashion and allowed us to confidently generate new single-cell datasets for two additional individuals. Combining results from the two methods allowed us to analyze nearly 100,000 unique paired αβ TCR sequences drawn from the CD4^+^ and CD8^+^ TCR repertoire of seven healthy individuals. In order to avoid introducing biases stemming from large clonal expansions, we will consider only the unique set of TCR sequences for each repertoire. We additionally note that the CD4^+^ and CD8^+^ repertoires may still be biased by the presence of many similar, but not identical, clones responding to the same viral epitope (26, 27). However, as each of these similar clones still must maintain its ability to recognize a particular MHC class and should represent a relatively small fraction of the repertoire in healthy individuals, the impact of these sequences on the observed repertoires is expected to be minimal.

Considering the unique set of TCR clonotypes (Vαβ and amino acid CDR3αβ) across all individuals, we found that the paired CD4^+^ and CD8^+^ repertoires were largely distinct from one another (αβ_*overlap*_ = 0.65% of total αβ sequences). Splitting the paired repertoire into the constituent α chain (α_*overlap*_=7.8%) and β chain (β_*overlap*_=4.7%) repertoires resulted in considerably higher overlap between the two lineages (Figure 1B). We note that α_*overlap*_ · β_*overlap*_ ≈ αβ_*overlap*_, potentially reflecting roughly independent contributions of the α and β chains. Quantifying the overlap between the CD4^+^ and CD8^+^ TCR repertoires within each individual, we observed greater similarity between the CD4^+^ and CD8^+^ single chain repertoires than between the paired αβ repertoires (Figure 1C). The decreased similarity of the paired TCR repertoires relative to the single chain repertoires, however, is not unique to the comparison of the CD4^+^ and CD8^+^ repertoires. For example, comparison of the single chain and paired CD4^+^ or CD8^+^ repertoires between individuals produces similar decreases in repertoire overlap and is likely reflective of the lower generation probability associated with a given αβ TCR pair relative to either of its constituent single chains.

Previous findings have suggested that TCRs shared between individuals may have shorter CDR3β sequences and may be closer to germline recombination sequences than clonotypes found only in a single individual (28). Accordingly, TCR sequences shared between the CD4^+^ and CD8^+^ lineages were, on average, shorter than those found only in one of the two lineages with respect to the α, β. and αβ repertoires (Figure 1D and Supplemental Figure 1). We further confirmed this finding using the OLGA software package to calculate the probability of randomly generating a given CDR3 sequence (25). As expected, TCR clones found in both repertoires additionally had higher generation probabilities than those found in a single repertoire (Figure 1E). Given the relative uniqueness of αβ TCRs for the CD4^+^ and CD8^+^ repertoires and previous structural findings implicating both chains in determining TCR-pMHC binding (7–10), we next asked whether αβ pairings could provide more information about T cell lineage than either chain alone.

###  Association of VJ Germline Segment Usage With CD4^+^-CD8^+^ Status

High-throughput sequencing of the β chain repertoire has revealed an association between the expression levels of specific TCR V-regions and MHC polymorphisms (29) and identified HLA-associated TCRβ sequences (30, 31). Furthermore, significant biases in V and J germline segment use between the single-chain CD4^+^ and CD8^+^ repertoires have been previously identified (22–24). One possible explanation for these observations, and generally a mechanism that enables MHC restriction, posits the existence of germline-encoded sequences that have been evolutionarily hard-wired into the Variable (V) region's CDR1 and CDR2 loops (32, 33). Evidence for such hard-wired regions biasing, but not completely determining, the interactions between TCRs and pMHC complexes is primarily drawn from a multitude of structural studies, which have identified a widely conserved TCR-MHC docking orientation (20), as well as other conserved TCR-MHC interaction motifs (34–38). The preference of specific germline regions for a particular MHC class is thought to create systematic biases in the CD4^+^ or CD8^+^ repertoires. We thus next hypothesized that if the paired αβ repertoire contained additional information about the function of the TCR repertoire, paired germline features should be more informative of T cell lineage than either of the single-chain repertoire alone. Specifically, information about αβ pairing should allow us to better understand the factors that influence TCR interaction with MHC and ultimately the factors at play in T cell differentiation.

**Figure 2:**
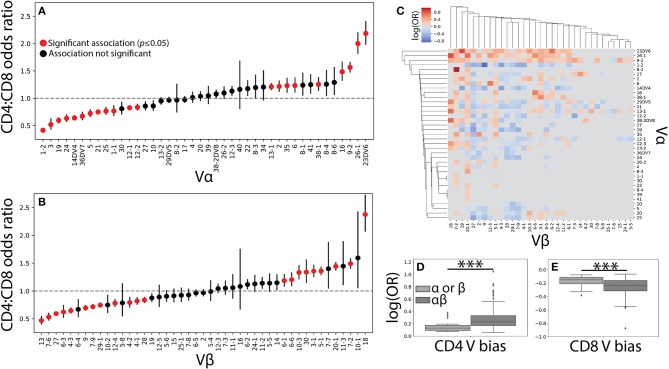
Vαβ pairings encode strong associations with T cell lineage. **(A)** The CD4^+^ and CD8^+^ TCR repertoires were then pooled across individuals and the CD4^+^:CD8^+^ odds ratio (OR) was calculated for each Vα and **(B)** Vβ germline region. An OR> 1 represents a CD4^+^ bias, while an OR< 1 represents a CD8^+^ bias with error bars representing the 95% confidence interval. The mean is represented by a red or black dot, with red representing statistical significance at *p*<0.05 by Fisher's exact test after Bonferroni correction. **(C)** Significant (q < 0.05 by Fisher's exact test) log odds ratios reveals strong CD4^+^:CD8^+^ biases for 349 Vαβ pairs. **(D)** Boxplots were calculated for the set of all significant odds ratios associated with single chains (Vα or Vβ) and compared with those associated with αβ pairs. Paired associations for both CD4^+^ and **(E)** CD8^+^ status were significantly stronger (^***^p < 0.001 by Mann-Whitney *U* test) than those for the single chain alone.

To further explore this possibility, we split the CD4^+^ and CD8^+^ repertoires into unique α, β, and αβ subsets, which allows us to directly compare each single-chain repertoire with that of pairs at similar sample sizes. We then calculated the odds ratio (OR) of observing a given Vα or Vβ in the CD4^+^ repertoire relative to the CD8^+^ repertoire. In this sense, the OR compares the odds of a given TCR feature being used in a given CD4^+^ TCR to the odds of it being used in a cell from the CD8^+^ population. Thus, an odds ratio that is >1 indicates a CD4^+^ bias, while an OR <1 is reflective of preferential use in the CD8^+^ repertoire. Calculating Bonferroni-corrected *p* values using the Fisher's Exact test, we identified weak, but statistically significant associations in both the Vα and Vβ single-chain repertoires (Figures 2A,B and Supplemental Figures 3A–D). Interestingly, these associations are significantly weaker than previously reported, potentially due to the more rigorous correction for PCR biases enabled by unique molecular identifiers (UMIs) available in single-cell sequencing (22). We further note weaker associations between T cell lineage and single chain Jα and Jβ usage (Supplemental Figures 2A,B, 3D–H).

The role of αβ germline segment pairing in biasing T cell differentiation was similarly examined by comparing the odds of observing a given Vαβ or Jαβ pair in each of the two repertoires. We show the statistically significant (*q* ≤ 0.05) CD4^+^:CD8^+^ odds ratios for 349 Vαβ and 79 Jαβ pairs associated with a significant lineage specification bias (Figure 2C and Supplemental Figure 2C). The strength of association with T cell lineage was significantly stronger for Vαβ pairs than for Jαβ pairs, likely reflecting the contribution of the CDR1 and CDR2 loops present in each V region to MHC binding (Supplemental Figures 2D–E). This finding supports the existence of germline-encoded TCR-MHC interaction motifs and raises the possibility that such motifs in both the α and β chains act in concert with one another.

Unsurprisingly, paired Vαβ provides a more nuanced view of germline associations when compared with the single-chain repertoires alone, with associations confined too specific pairs (Figure 2C). Qualitatively, our data reveals several associations in the paired data that would have otherwise been missed in the single chain results. For example, TRBV20-1 is strongly associated with CD4^+^ status in the single chain dataset, but paired analysis reveals several α chains for which TRBV20-1 has significant CD8^+^ associations (e.g., TRAV1-2, TRAV19, TRAV36DV7). Similarly, TRAV4 has no association in the single chain data, but several associations with specific β chains (e.g., TRBV6-5, TRBV5-1, TRBV2). The observed associations between paired Vαβ germline regions and T cell lineage were additionally, on average, significantly stronger than those associations found for either of the single chain repertoires individually (Figures 2D–E). Biologically, this finding is consistent with the notion that both the α and β chain contribute substantially to TCR-pMHC binding (7–10). Furthermore, these germline region biases are observed across individuals of differing HLA types and are consequently likely to be representative of differences between MHC classes rather than from individual MHC polymorphisms.

###  CDR3 Features Alone Are Weakly Associated With T Cell Lineage

**Figure 3:**
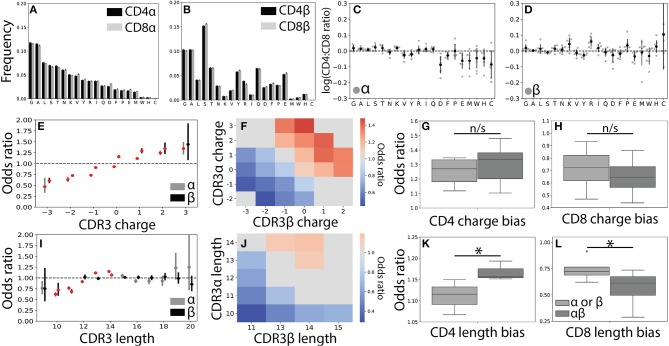
CDR3 features correlate weakly with T cell lineage. **(A)** Usage frequencies for all 20 amino acids, rank ordered by prevalence in CDR3α, are shown for CDR3α and **(B)** CDR3β sequences across the CD4^+^ and CD8^+^ repertoires. **(C)** The CD4^+^:CD8^+^ usage ratio for all amino acids are shown for the α and **(D)** β chains. The frequency with which each amino acid is used is shown for each individual (gray circles) with the population mean and standard deviation shown in black. Amino acid usage was found to not significantly differ across the CD4^+^ and CD8^+^ repertoires using a one sample *t*-test after Bonferroni correction. **(E)** CD4^+^ odds ratios (OR) quantify the strength of association of CDR3 net charge with lineage for both the α (gray, left) and β (black, right) chains. Red markers indicate statistical significance (*p* < 0.05 after Bonferroni correction). **(F)** Significant (*p* < 0.05 after Bonferroni correction) log odds ratios reveals strong CD4^+^:CD8^+^ bias for 23 CDR3αβ charge pairs. Values that are not statistically significant are shown in gray (OR defined as being equal to one). **(G)** Boxplots compare the strength of association between T cell lineage and either single-chain features (α or β) and paired (αβ) CDR3 charges. Paired charges show stronger associations when compared with those of the single-chain for both the CD4^+^ and **(H)** CD8^+^ populations. **(I)** Single-chain odds ratio associations for CDR3 length. **(J)** Significant paired CDR3αβ length association with T cell lineage. As in **(F)**, only statistically significant CD4^+^:CD8^+^ odds ratios are shown. **(K)** Boxplot compares length association strength with CD4^+^ or **(L)** CD8^+^ status for paired and single-chain features. ^*^p < 0.05 by Mann Whitney *U*.

Conventionally, the CDR1 and CDR2 loops encoded entirely within the germline Vα and Vβ regions have been thought to predominate the TCRs interaction with MHC. However, recent structural evidence has additionally noted interactions between the CDR3 region, which predominantly drives antigen specificity, and MHC (20, 21). As such, we additionally investigated the role of CDR3αβ pairing in driving MHC class I or II recognition. To gain a better understanding of CDR3 composition, we first calculated the frequency with which each amino acid was used across all α or β CDR3 regions. We observed strong differences in amino acid usage between the α and β chains, likely due to differences in the germline composition of α and β V(D)J segments (Figures 3A,B). However, we observed only small, insignificant differences in amino acid use between the CD4^+^ and CD8^+^ repertoires (Figures 3C,D). Although the overall effect size remained small, we did note increased use of negatively charged amino acids in the CD8^+^ T cell population in both the α and β repertoires. Similarly, our data suggested an increased use of positively charged amino acids in the CD4^+^ population.

In order to gain a better understanding of how CDR3 net charge may effect MHC recognition, we calculated the odds ratio for CDR3 net charge between the two T cell populations. As expected (22, 23), we found that net positive charges were significantly associated with CD4^+^ status and net negative charges were associated with the CD8^+^ population (Figure 3E and Supplemental Figures 4A–D). We next calculated the odds ratio for joint CDR3αβ charge pairs, finding a similar pattern to the single chain data (Figure 3F). Associations between T cell lineage and CDR3 charge were stronger for the paired chains, though these differences not statistically significant for CDR3 charge (Figures 3G,H).

To further explore the relationship between the CDR3 region and T cell lineage, we next examined CDR3 length. As found in previous studies, we identified only very weak relationships between lineage and CDR3α and CDR3β lengths (Figure 3I and Supplemental Figures 4E–H) (22, 23). Interestingly, however, we do observe a small number of CDR3αβ length pairs that have significant associations with CD4^+^ and CD8^+^ status (Figure 3J). Again, we find that these paired interactions are substantially stronger than those found in the single-chain repertoire (Figures 3K,L). We note that associations for CDR3 charge and length were substantially weaker than those identified for Vαβ pairs, consistent with CDR3 sequence playing a smaller role in the TCR-MHC interaction than germline regions.

###  Paired Chain Sequences Are More Informative of CD4^+^-CD8^+^ Status Than Single Chains

**Figure 4:**
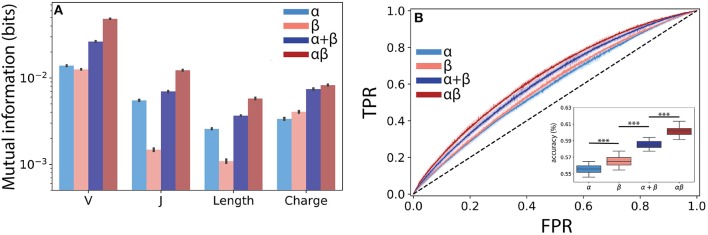
Paired αβ sequences are more informative of T cell lineage than single chain sequences alone. **(A)** Mutual information estimates (bits) were calculated using a finite-sampling correction to quantify the amount of information about T cell lineage by various TCR features drawn from the α, β, α and β summed together (α + β), and paired αβ repertoires. α + β sum represents expected mutual information if contributions from each chain are conditionally independent of T cell lineage. **(B)** A boosted tree classifier [XGBoost (40)] was trained using a constant length vector encoding V and J region usage, CDR3 length, and CDR3 amino acid usage frequencies. Receiver operating characteristic (ROC) curves showing true positive rate (TPR) vs. false positive rate (FPR) for the classifiers trained using the α and β single chain and the αβ paired repertoire. To determine the expected accuracy of a classifier trained using independent α and β chains, we created a soft-voting classifier trained on each chain individually (α + β). Inset shows prediction accuracy for classifiers trained using data from ECH repertoire. Shaded region represents one standard deviation. Boxplot inset show maximum accuracy in predicting CD4^+^ or CD8^+^ from each repertoire through 5-fold cross-validation repeated 10 times. Statistical significance between groups was assessed by Mann-Whitney *U* test (^***^p < 0.001).

To quantify the amount of information about CD4^+^ and CD8^+^ status encoded in the α, β, and αβ TCR sequences, we next calculated the mutual information (39), corrected for finite sample sizes, between several TCR features and T cell lineage (Figure 4A). In brief, mutual information allows us to quantify the dependence of two random variables (e.g., the dependence of CD4^+^/CD8^+^ status on Vα gene usage), with a mutual information value of zero corresponding to statistical independence. Examining single chain V and J germline region usage frequencies, as well as CDR3 length and charge distributions, we find a small but non-negligible amount of information about T cell lineage. If the α and β chains encode information about T cell lineage in a conditionally independent manner, the expected information content of αβ pairs can be found by summing the information contained by each chain individually (α+β). Alternatively, the α and β could encode redundant information (as would be the case if the β chain was the predominate determinant of TCR-pMHC interactions) and would result in the information contained in αβ pairs being less than the sum of the two chains (αβ < α + β). Surprisingly, we observe synergistic information (41) in which the paired chains carry more information than the individual chains summed together (αβ > α + β), which would suggest that the germline encoded interactions may act in a synergistic manner and highlights the importance of both chains in determining TCR function. Despite this observed synergy, the overall amount of information encoded in these general TCR features about T cell lineage remains relatively low.

Building from this observed synergistic information built into αβ pairings, we next asked whether the use of paired sequences would better allow us to predict T cell lineage from TCR features using machine learning classification. We obtained the highest accuracy using a gradient boosted decision tree classifier, specifically the XGBoost (40) algorithm (see Methods). Although the α (AUC ≈ 0.59 ± 0.006) and β (AUC ≈ 0.60 ± 0.006) chains were both only weakly informative of lineage, we found that the information encoded by paired TCR sequences (αβ AUC ≈ 0.64 ± 0.005) allowed for a significant increase in model performance (Figure 4B). As our mutual information calculations demonstrated the presence of synergistic information within αβ pairings, we reasoned that our machine learning classifier should reflect this additional information. To address this question, we independently trained classifiers on both the α and β chains and created a soft-voting ensemble (α + β) to predict CD4^+^ and CD8^+^ lineage. Classifiers trained on αβ pairs together significantly outperformed those trained on the additive model (additive AUC≈ 0.63±0.004, *p* ≤ 3 × 10^−15^ against αβ using a Mann Whitney *U* test), again suggesting a synergistic relationship between α and β pairs with respect to T cell lineage specification (Figure 4B inset).

Of note is a previous report using a SVM classifier and CDR3 length-dependent parametrization to predict T cell lineage from TCR sequences with >90% accuracy (23). This approach, however, failed to achieve the same degree of predictive accuracy when using our dataset (Supplemental Figure 5). To better understand this finding, we compared the TCR sequences from this previous study (23) with those reported here and an additional bulk-sequencing TCRβ dataset (24). We find that the aforementioned increased predictive accuracy is driven by anomalous Vβ and Jβ gene frequencies in the Li et al. dataset, possibly due to a lack of rigorous PCR correction, as compared with the other two datasets (Supplemental Figure 6).

###  Association of Paired αβ Sequences With Known Peptide Specificity

Given the increased information contained within paired αβ TCR sequences about T cell lineage, we next asked whether these paired sequences could provide us with additional information about peptide specificity. More specifically, we wondered whether information from αβ pairing could be used to significantly improve our ability to understand the functional aspects of the TCR repertoire. In order to address this question, we downloaded more than 20,000 CDR3 sequences with known antigen specificities from a previously published repository [VDJdb (42)]. Of these known TCRs, more than ~96% were known to recognize peptides presented by MHC class I, with the remaining ~4% recognizing peptides presented in the context of an MHC class II molecule. We note that the antigen annotations provided for the curated VDJdb TCR sequences were obtained experimentally, most frequently through tetramer sorting assays (42).

**Figure 5:**
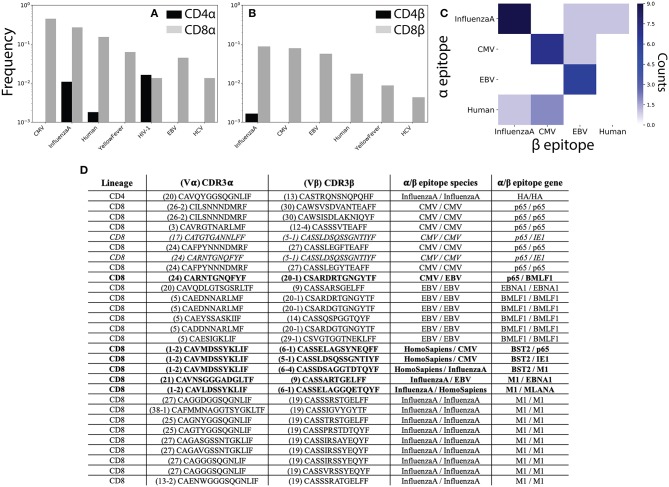
αβ pairing provides additional information about antigen specificity. **(A)** The VDJdb (42) database of TCRs with known peptide specificity was compared to the CD4^+^ and CD8^+^ single-chain repertoires for the α and **(B)** β chains. **(C)** Antigen specificities for paired αβ TCR sequences. Counts along the diagonal represent pairs with matching specificity while those off the diagonal represent mismatched pairs. **(D)** Table shows CDR3 sequence and antigen specificity for all αβ pairs for which annotations were available for both chains. TCR pairs for which the α and β chains recognize epitopes from different species are shown in bold, while those recognizing different epitopes from the same species are shown in italics. CMV, cytomegalovirus; EBV, Epstein-Barr virus; HIV, human immunodeficiency virus; HCV, hepatitis C virus; HTLV, human T-lymphotropic virus; DENV, Dengue virus.

We first compared our single chain CD4^+^ and CD8^+^ TCR repertoires against these known sequences, using clonotypes composed of the V region plus amino acid CDR3 sequence, reporting the fraction of each repertoire with known antigen annotations (Figures 5A,B). In total, we identified 287 α and β TCR sequences with experimental antigen specificity annotations, of which 17 (~5%) were found in the CD4^+^ repertoire (in line with the 4% of VDJdb annotations corresponding to MHC II restricted epitopes). Of these sequences, ~80% of α and β chains were associated with highly prevalent viral infections (Cytomegalovirus, Epstein-Barr virus, Influenza A) to which public TCR clones have previously been observed in otherwise healthy individuals (43). Interestingly, the remaining 20% of annotated sequences recognized epitopes that should not be present in our healthy cohort (e.g., Yellow Fever, HIV, and Hepatitis C). Of note, this result further demonstrates the ability of single-cell sequencing (17, 18) approaches to capture large numbers of TCRs which have previously been observed using bulk-sequencing methodologies.

To better understand how analysis of αβ paired TCR sequences would influence our ability to understand TCR antigen specificity and repertoire-level function, we next asked which of our αβ pairs had known peptide specificities for both the α and β chains individually. We observed 1 CD4^+^ and 28 CD8^+^ TCR pairs for which for which both chains had known antigen specificities. Of these, 6 (~21%) TCR pairs recognized epitopes from different species and an additional 2 (~7%) pairs recognized different epitopes from the same species (Figures 5C,D). In contrast to the single-chain repertoires, all TCR pairs with matching antigen specificities recognized a viral antigen expected to be found in healthy individuals (Figure 5C). We additionally note several non-monogamous αβ pairings in which the same α chain is paired with β chains recognizing different antigens. For example, the α sequence Vα1-2 CAVMDSSYKLIF has previously been shown to recognize a human Bone Marrow Stromal Cell Antigen 2 (BST2) epitope and is here shown to pair with β sequences that have been shown to interact with both Influenza A and CMV epitopes (Figure 5D).

While promiscuity in TCR pairing has been widely reported (19, 26, 44, 45), these results serve to further emphasize the functional importance of αβ pairing in determining antigen specificity. That is, our findings clearly demonstrate the ability of an identical TCR sequence to recognize differing pMHC complexes depending on its pair. Given recent efforts to infer antigen exposure history from the β chain repertoire (24), the prevalence of antigen false-positives observed in the single chain repertoires (i.e., TCRs recognizing antigens not present in healthy donors) may be of particular relevance. Further, these findings demonstrate that even limited sequencing of the paired αβ repertoire may be able to provide accurate information about previous antigen exposure and repertoire function.

## Discussion

Although the theoretical importance of αβ pairing is not debated, the actual amount of functional information which can be extracted from repertoire level analyses of αβ TCR pairs remains uncertain. In this study, we have contributed more than 11,000 unique αβ paired sequences to our previously published database, providing us with nearly 100,000 unique TCR pairs split between the CD4^+^ and CD8^+^ T cell lineages. To better understand how high-throughput examination of αβ pairing can inform on repertoire function, we chose to focus on (i) how TCR pairing might influence MHC recognition and subsequently inform on biases between the CD4^+^ and CD8^+^ repertoires, and (ii) how TCR pairing might provide additional information on the antigen specificity of the TCR repertoire.

A growing number of studies have begun to elucidate a number of molecular interactions conserved between multiple structures leading to the hypothesis that such interaction motifs have been evolutionarily incorporated into the germline Vαβ sequences (32, 33). Although these conclusions are primarily based on a limited number of solved TCR-pMHC structures, bulk-sequencing of the β chain has revealed statistical associations between features of the TCR repertoire and individual MHC polymorphisms (29). However, previous studies have not differentiated between the CD4^+^ and CD8^+^ paired TCR repertoires. It was therefore unknown whether αβ pairing could influence the effects of these germline biases. Our analysis of the healthy CD4^+^ and CD8^+^ TCR repertoires revealed that while individual α and β chains were more commonly found in both repertoires, paired αβ sequences tended to be specific for one lineage. As has been previously suggested for β chains (28), we found that sequences shared between the two cell lineages tended to be shorter and have higher generation probabilities (i.e., are closer to the germline sequences) than those found only in one repertoire. Together, these results suggested that a large portion of paired αβ sequences were relatively specific for one MHC class and supported previous findings of systematic differences between the CD4^+^ and CD8^+^ repertoires (22–24).

Comparing the α and β single-chain repertoires between the CD4^+^ and CD8^+^ expectedly revealed that V and J germline region usage, as well as CDR3 charge and length distributions, differed between the two repertoires. Consistent with previous small-scale structural findings (7–10), our results demonstrate that αβ pairings encode substantially stronger associations with T cell lineage than either of the single-chain repertoires alone. Rigorously quantifying the strength of these associations using mutual information and machine learning classifiers, our results showed that the majority of information about T cell lineage carried by TCRs is encoded by the V germline region, with significantly less information present in the J region, CDR3 charge, and CDR3 length. Though the total amount of information remained relatively low, these methods revealed substantial synergy between the α and β chains with respect to lineage association. To the best of our knowledge, such synergy between TCR chains, particularly for Vαβ pairs, has not been previously demonstrated at the repertoire level. Biologically, one possible explanation of these findings is a model in which Vα and Vβ chains evolved to, in concert, bias TCRs toward interaction with either of the MHC classes. Future studies employing a substantially larger cohort will be necessary to further unravel the relationship between specific TCR features and HLA-types, as well as specific MHC polymorphisms.

Finally, given the observed importance of αβ pairing in driving MHC specificity, we asked whether TCR pairings could similarly influence peptide specificity. Toward this, we annotated our TCR sequences using the antigen specificity information contained within the VDJdb sequence repository (42). We found that approximately one third of our TCR pairs for which both chains had known antigen specificities were mismatched (i.e., had different known antigen specificities for the α and β chain). Intriguingly, TCR pairs recognizing the same antigen individually were always associated with common viral peptides that would be expected to be present in otherwise healthy individuals. Conversely, TCR pairs with different antigen specificities tended to recognize viral peptides that are not found in healthy individuals, suggesting that the antigen specificity of single chains is largely dependent upon its pair. This result is consistent with previous findings from bulk-sequencing of the β chain in CMV patients, in which even CMV seronegative patients were found to have low levels of CMV-associated TCRs (24). While this study was largely successful in predicting whether an individual was infected with CMV from the TCRβ repertoire, it required the use of a large number of TCR sequences with known CMV associations. Given the demonstrated increase in antigen specificity information contained within αβ pairing, we hypothesize that the increased availability of such single-cell approaches may ultimately increase diagnostic efficiency and accuracy.

In summary, we have generated and comprehensively analyzed the largest database of CD4^+^ and CD8^+^ paired αβ TCR sequences to date using recently developed high-throughput single-cell technologies. While such single-cell methods remain cost-prohibitive for large cohort studies, we have demonstrated the ability of current paired αβ sequencing to provide useful insights into TCR repertoire function beyond those available from conventional bulk-sequencing. Biologically, our results have shown substantial synergistic information about T cell lineage encoded within TCR pairings and suggested the utility of αβ pairings when determining antigen specificities for an individual's TCR repertoire. Together, our results demonstrate the power of paired αβ sequencing to inform on repertoire function and suggest that current paired αβ repertoire sequencing are capable of opening new avenues of research when use in conjunction with TCRβ sequencing. We further believe that the rigorous examination of the normal αβ TCR repertoires presented in this study will prove to be valuable in understanding the perturbations caused by infectious, oncological and autoimmune disease states.

## Materials and Methods

###  Single-Cell Barcoding and Sequencing

TCR sequences for Subjects 1-5, along with each patient's HLA type, were obtained from Grigaityte et al. (19). As described previously, peripheral blood mononuclear cells (PBMCs) were obtained from five healthy donors after obtaining appropriate informed consent. Blood samples underwent pan T cell enrichment before single-cell barcoding-in-emulsion using the AbVitro microfluidic platform (18). In brief, single-cell sequencing was performed by probabilistically loading individual T cells into ~65 picoliter oil-emulsion droplets and TCR-targeted reverse-transcriptase PCR is performed. Unique droplet barcodes, along with unique molecular identifier (UMI) barcodes, are similarly loaded into droplets and attached to TCR cDNA within each droplet. Droplets are then lysed and next-generation sequencing performed on the pooled product using the Illumina MiSeq platform (18). Raw sequences were processed using a custom pipeline (19) to identify αβ pairs utilizing MiXCR 2.2.1 (46) to identify V(D)J segments and annotate the CDR3 region of each TCR. As described in detail previously (19), the quality of TCR pairs were ensured by setting a minimum read depth for including a given TCR sequence and collapsing reads from a single droplet with a nucleotide CDR3 Hamming distance of 1. We excluded droplets with more than one unique α or β chain given that we cannot readily differentiate droplets with an allelic inclusion T cell from those containing two different T cells.

All TCR sequences for Subject 6 and 7, as well as for a subset of Subjects 1 and 3, were obtained using the 10× Genomics commercial single-cell sequencing platform (17). PBMCs for Subjects 6 and 7 were purchased from ATCC (PCS-800-011TM). CD4^+^ and CD8^+^ T cell populations were separated using either magnetic bead enrichment according to the manufacturer protocol (EasySep Human T Cell Enrichment Kit, StemCell Technologies) or fluorescence activated cell sorting (Becton Dickinson FACSARIA SORP). Following the manufacturer's instructions, ~5,000 cells per lane were loaded into the Chromium Controller using the Single Cell V(D)J reagent kit for emulsion-barcoding (17) and sequenced using an Illumina HiSeq 2500 sequencer. Raw sequencing reads were processed as described above (19). PBMC samples for Subjects 1 and 3 used for sequencing on the 10× platform were frozen and stored for several months, potentially leading to RNA degradation and resulting in the low number of captured sequences.

The Li et al. dataset (23) was provided by N. P. Weng as a processed datafile containing VJ segments and CDR3 amino acid sequences. The Emerson et al. dataset (24) was downloaded from Adaptive Biotechnologies open-access immuneACCESS database (https://clients.adaptivebiotech.com/immuneaccess). While healthy and diseased TCR repertoires were obtained, only the 17 healthy patients were studied here.

###  Data Analysis

Paired αβ TCR sequences, along with clonotype information about V(D)J segment use and CDR3 amino acid sequences, were divided into CD4^+^ and CD8^+^ repertoires. T cells lacking a lineage designation or expressing two unique TCRs (i.e., dual receptor T cells) were excluded from subsequent analysis. As we care about identifying features of the TCR repertoires between the CD4^+^ and CD8^+^ populations, we count each unique TCR clonotype only once. That is, clonal expansion in the CD4^+^ and CD8^+^ populations would bias our analysis of the factors that affect differentiation. As such, we include each TCR clonotype only once into our final dataset. We then identified TCR clonotypes that were shared between the CD4^+^ and CD8^+^ compartments and the degree of overlap between the two TCR repertoires was quantified using the Jaccard Index (*J*):

(1) $$\begin{array}{r} J\left(CD4,CD8\right)=\frac{\left|CD4\cap CD8\right|}{\left|CD4\cup CD8\right|} \end{array}$$

Here |*CD*4 ∩ *CD*8| refers to the cardinality of the intersection between the CD4^+^ and CD8^+^ TCR repertoires (i.e., the number of TCRs found in both repertoires). |*CD*4 ∪ *CD*8| refers to the union of the two repertoires (i.e., the number of TCRs found in either of the two repertoires). The Jaccard Index was calculated independently for the α (*J*(*CD*4_α_, *CD*8_α_)), β (*J*(*CD*4_β_, *CD*8_β_)), and αβ (*J*(*CD*4_αβ_, *CD*8_αβ_)) TCR repertoires.

Furthermore, as done previously (19), the paired αβ repertoire consists of all unique, paired TCR sequences and the α and β individual chain repertoires were derived directly from the paired repertoire. That is, the individual α repertoire consists of all the α chains present in the paired dataset. Thus, the α, β, and αβ datasets are all of the same size and differences in sample size do not drive the observed differences. Furthermore, all boxplots represent median and inter-quartile range.

###  VJ Segment Usage

V(D)J segments were identified from raw sequences by MiXCR and annotated according to the International ImMunoGeneTics (IMGT) V(D)J gene definitions (47). The odds ratio (OR) for a given TCR characteristic and T cell lineage was calculated by counting the number of TCRs with (*C*^+^) and without (*C*^−^) that characteristic within the CD4^+^ (*T*^4^) and CD8^+^ (*T*^8^) repertoires. The OR is then given as:

(2) $$\begin{array}{r} OR=\frac{\left|C^{+}\in T^{4}|*|C^{-}\in T^{8}\right|}{\left|C^{-}\in T^{4}|*|C^{+}\in T^{8}\right|} \end{array}$$

The numerator is the number of CD4^+^ TCRs with a given feature multiplied by the number of CD8^+^ TCRs without that feature. The denominator is given by the number of CD4^+^ cells without that feature multiplied by the number of CD8^+^ with that feature. 95% confidence intervals and a *p*-value were then calculated for each OR using Fisher's exact test implemented using the SciPy library (www.scipy.org). Multiple hypothesis testing correction was applied to single chain *p*-values using a Bonferroni correction and paired chains *p*-values, given the larger number of tested hypotheses, were converted to *q*-values (48). Significance was assessed at the *p* < 0.05 or *q* < 0.05 level.

###  CDR3 Features

Sequence logos showing the amino acid frequency for a given position in the sequence were generated using all α and β CDR3 sequences of length 14 using WebLogo (49). Of note, we defined the CDR3 length to be inclusive of the proximal cysteine and terminal phenylalanine that define the CDR3 region. The ratio of each amino acid in CDR3 between each population was calculated by dividing the frequency of a given amino acid across all CD4^+^ CDR3 sequences for a given chain by the frequency with which that amino acid occurred across all CD8^+^ CDR3 sequences. CDR3 charge was calculated as difference between the number of positively charged amino acids (R and K) and negatively charged amino acids (D and E) present in the CDR3 region.

###  Mutual Information

The mutual information (I, bits), between a given feature, X, and T cell lineage (L) was calculated as:

(3) $$\begin{array}{r} I\left(X;L\right)=\underset{x\epsilon X}{\sum }\underset{l\epsilon L}{\sum }p\left(x,l\right)\log_{2}\left(\frac{p\left(x,l\right)}{p\left(x\right)p\left(l\right)}\right) \end{array}$$

In order to correct for biases in our MI estimate arising from our limited sample sizes, we applied a bootstrapping based finite-sampling correction previously described (19, 50). We additionally calculate the synergistic information (41) (*S*) according to:

(4) $$\begin{array}{r} S\left(X_{\alpha },X_{\beta },L\right)=I\left(X_{\alpha },X_{\beta };L\right)-I\left(X_{\alpha };L\right)-I\left(X_{\beta };L\right) \end{array}$$

where *X*_α_ and *X*_β_ refer to TCRα and TCRβ features, respectively.

###  Machine Learning

Extreme Gradient Boosted decision tree classifiers were trained using the Python XGBoost implementation (40). α and β chain TCR sequences were converted into length-independent vectors encompassing V and J regions (categorically encoded), CDR3 length, CDR3 charge, and CDR3 amino acid usage frequencies. Only the unique set of TCRs were used for training and testing, and TCR sequences found in both the CD4^+^ and CD8^+^ repertoires were removed. Classifiers were trained and tested using 5-fold cross-validation, which was repeated 10 times, for the each of the α, β, or αβ repertoires. The independent α+β classifier was an ensemble classifier created by training XGBoost classifiers on each chain independently. Final predictions for this ensemble were made using a soft-voting approach. Receiver operating characteristic curves (ROC), as well as the area under the ROC curve (AUC) and classifier accuracy, were calculate using the sklearn metrics package (51). ROC curves and AUC values were calculated using the predicted probability of a given TCR chain belonging to the CD4^+^ population. Accuracy was calculated as the percentage of correct predictions divided by the total number of predictions made, where a TCR was predicted to be CD4^+^ sequence if the predicted CD4^+^ probability was >50%. For SVM's trained on the Li et al. and Emerson et al. dataset, CDR3β amino acid sequences were first converted in numeric vectors using Atchley factors (23, 52).

## Data Availability

Sequencing data and custom Python scripts used for data analysis are freely available at our Github Repository (https://github.com/JasonACarter/CD4_CD8-Manuscript).

## Author Contributions

JC and GA contributed to the conception and design of the study. JC, JP, KG, SG, and EJ performed research with supervision from AB, FV, and GA. JC and GA analyzed data and wrote the paper. All authors contributed to manuscript revision, read, and approved the submitted version.

### Conflict of Interest Statement

SG, EJ, and AB are employed by Juno Therapeutics and hold equity in its parent company, Celgene. FV was formerly employed by Juno Therapeutics and is currently employed by and holds equity in Shape Therapeutics. The remaining authors declare that the research was conducted in the absence of any commercial or financial relationships that could be construed as a potential conflict of interest.

## Supplementary Material

Supplemental Figure 1 CDR3 sequences shared between the CD4^+^ and CD8^+^ repertoires tend to be shorter than those found in only one repertoire. CDR3 length distributions show sequences found in both the CD4^+^ and CD8^+^ repertoires (∩) are shorter than those found in only one of the two repertoires (⊕) for the **(A)** α, **(B)** β, and **(C)** paired αβ repertoires. For paired sequences, we report the average length of the α and β chains. **(D)** Heatmaps showing frequency with which each α and β CDR3 length pair is present in the TCR repertoire shared between the CD4^+^ and CD8^+^ lineages and for the **(E)** TCR repertoire present in only one of the two lineages. Dashed red lines indicate the average length for the α (14 amino acids) and β chains (15 amino acids).

Supplemental Figure 2 J germline region bias for the α, β, and αβ repertoires. **(A)** The CD4^+^ and CD8^+^ TCR repertoires were then pooled across individuals and the CD4^+^:CD8^+^ odds ratio (OR) was calculated for each Jα and **(B)** Jβ single-chain germline region. An OR>1 represents a CD4^+^ bias, while an OR <1 represents a CD8^+^ bias with error bars representing the 95% confidence interval. The mean is represented by a red or black dot, with red representing statistical significance at the *p* < 0.05 by Fisher's exact test level after applying Bonferroni correction. **(C)** Significant (*q* < 0.05 by Fisher's exact test) log odds ratios reveals strong CD4^+^:CD8^+^ biases for 79 Jαβ pairs. **(D)** Boxplots were calculated for the set of all significant odds ratios associated with single chains (Jα or Jβ) and compared with those associated with Jαβ pairs. Paired associations for both CD4^+^ and **(E)** CD8^+^ status were significantly stronger (^***^*p* < 0.001 by Mann-Whitney *U* test) than those associated with a single chain alone. Associations for the J region were, overall, substantially weaker than those observed for the V chain.

Supplemental Figure 3 V and J germline region usage. **(A)** Single-chain V region distributions for the α and **(B)** β chains. **(C)** Paired Vαβ usage for the CD4^+^ and **(D)** CD8^+^ T cell populations. **(E)** Single-chain J region distributions for the α and **(F)** β chains. **(G)** Paired Vαβ usage for the CD4^+^ and **(H)** CD8^+^ T cell populations.

Supplemental Figure 4 CDR3 charge and length distributions. **(A)** Single-chain CDR3 charge for the α and **(B)** β chains, separated by CD4^+^ and CD8^+^ populations. **(C)** Paired CDR3αβ charge usage for the CD4^+^ and **(D)** CD8^+^ T cell populations. **(E)** Single-chain CDR3 length for the α and **(F)** β chains, separated by CD4^+^ and CD8^+^ populations. **(G)** Paired CDR3αβ length distributions for the CD4^+^ and **(H)** CD8^+^ T cell populations.

Supplemental Figure 5 SVM trained on CDR3β sequences converted to Atchley factors. A support vector machine (SVM) was trained on vectors composed of CDR3β sequences converted into numerical array according to their Atchley factors. As these vectors are dependent on the length of the CDR3 sequence, SVMs were trained separately for CDR3 sequences of lengths between 10 and 15, as previously done (23). For comparison, SVM accuracy for classifiers trained on CDR3β sequences converted to our constant length vector are also shown (Constant). **(A)** Accuracy for each model is reported as the percentage of correctly predicted CDR3 sequences using an independent testing set (25% of dataset). The Li et al. dataset is well-described by this SVM model, with accuracy as high as 96%. However, this model fails to accurately describe either the dataset used in this study or that of Emerson et al. **(B)** Receiver operator curves (ROC) for the current dataset, **(C)** the Emerson et al. dataset, and **(D)** the Li et al. dataset show length-dependent SVMs accurately predict the Li et al. dataset, but fail to do so for the other two datasets.

Supplemental Figure 6 V and J region usage patterns vary substantially between the Li et al. and Emerson et al. datasets. **(A)** β TCR sequences were obtained from 621,085 CD4^+^ and 64,725 CD8^+^ cells previously by Li et al. (23). Comparison of V-usage frequencies for each germline region reveals large differences between the CD4^+^ and CD8^+^ repertoires in this dataset. **(B)** V-usage frequencies observed by comparing 3,212,682 CD4^+^ and 1,774,260 CD8^+^ TCR sequences taken from Emerson et al. reveal less variation between the two cell types (24) and more closely resemble the results obtained in the present study (Supplemental Figure 3). **(C)** Similar results were obtained for Jβ region usage in the Li et al. and **(D)** Emerson et al. datasets. **(E)** We quantified the difference in V segment use in the CD4^+^ and CD8^+^ populations by calculating the odds ratio (OR) for each V region in the Li et al. dataset and **(F)** the Emerson et al. dataset independently. **(G)** Jβ usage between Li et al. dataset and **(H)** Emerson et al. datasets. **(I)** Mutual information with finite sampling correction was calculated for the association between β chain features (Vβ, Jβ, CDR3β length and charge) and lineage for the dataset used in this study (from Table), by Li et al. (23) and Emerson et al. (24). Substantially higher mutual information values, indicating stronger associations, were found for the Li et al. dataset as compared to the other two datasets.

Supplemental Table 1 Demographic information for each subject. Peripheral blood mononuclear cells (PBMCs) were previously obtained from 5 healthy individuals (S1–S5) and sequenced using single-cell barcoding in emulsion (18, 19). The original PBMC samples from S1 and S3, as well as new samples from additional healthy individuals (S6, S7), were sequenced using a commercially available single-cell system (10× Genomics) (17). In all, we obtain 70,108 and 26,946 unique TCR pairs from CD4^+^ CD8^+^ T cells, respectively. Demographic information, as well as HLA types, are provided as available (19).
