# Supplementary figures and images for: Single T Cell Sequencing Demonstrates the Functional Role of αβ TCR Pairing in Cell Lineage and Antigen Specificity
Source: Front Immunol. 2019 Jul 31;10:1516. doi: 10.3389/fimmu.2019.01516 (PMC6684766; doi:10.3389/fimmu.2019.01516)

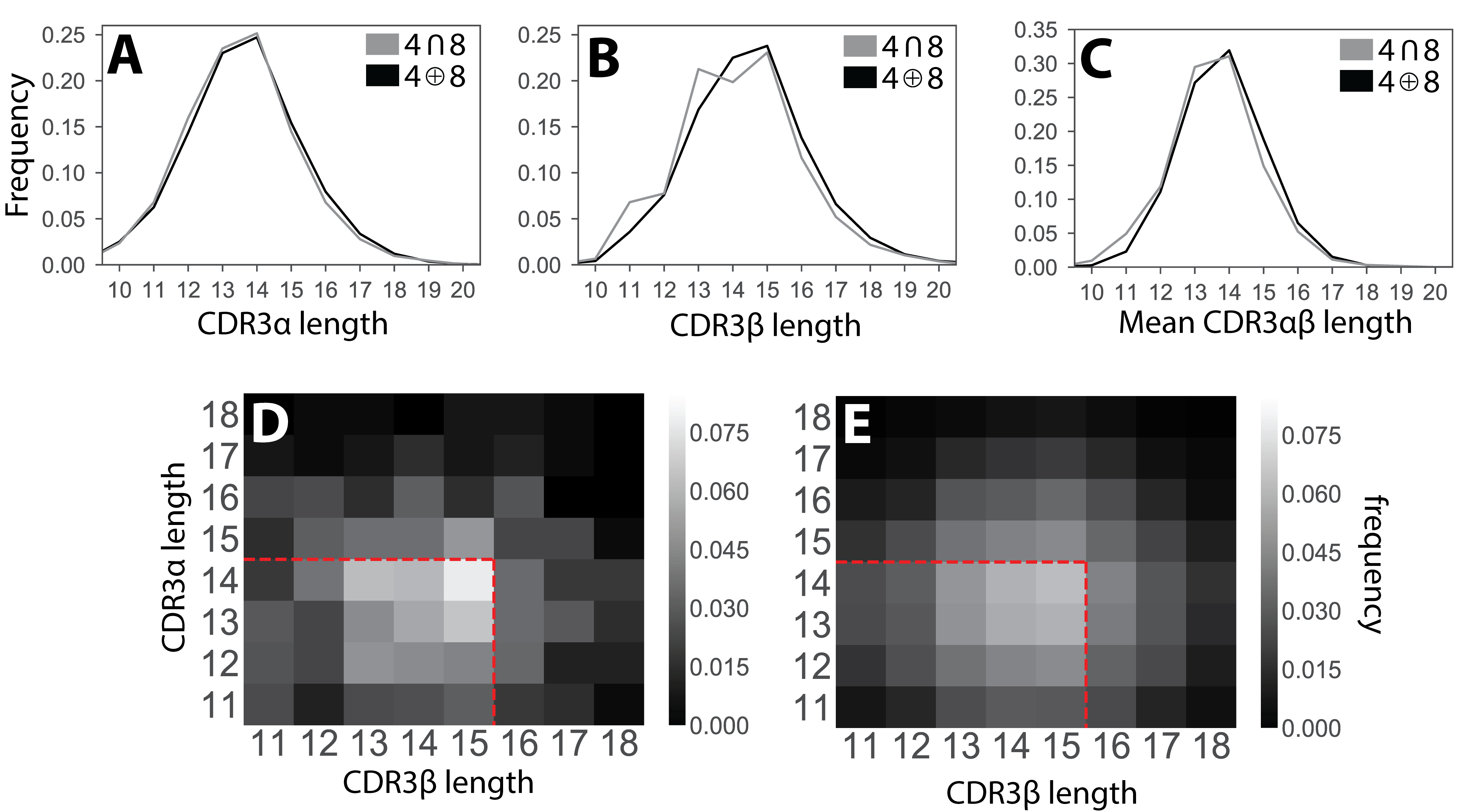

Supplement: Supplemental Figure 1 — CDR3 sequences shared between the CD4+ and CD8+ repertoires tend to be shorter than those found in only one repertoire. CDR3 length distributions show sequences found in both the CD4+ and CD8+ repertoires (∩) are shorter than those found in only one of the two repertoires (⊕) for the (A) α, (B) β, and (C) paired αβ repertoires. For paired sequences, we report the average length of the α and β chains. (D) Heatmaps showing frequency with which each α and β CDR3 length pair is present in the TCR repertoire shared between the CD4+ and CD8+ lineages and for the (E) TCR repertoire present in only one of the two lineages. Dashed red lines indicate the average length for the α (14 amino acids) and β chains (15 amino acids). [file Image_1.JPEG]

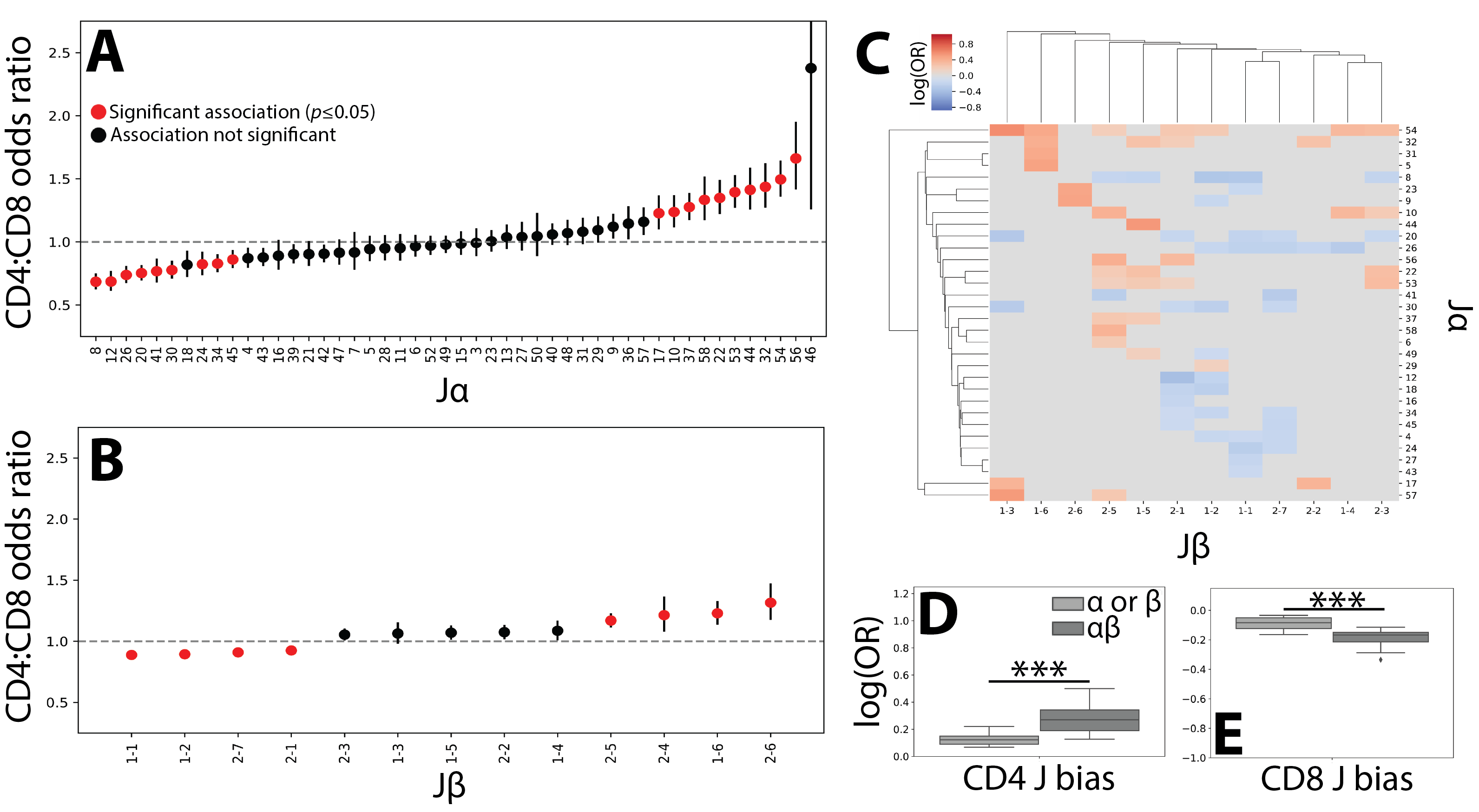

Supplement: Supplemental Figure 2 — J germline region bias for the α, β, and αβ repertoires. (A) The CD4+ and CD8+ TCR repertoires were then pooled across individuals and the CD4+:CD8+ odds ratio (OR) was calculated for each Jα and (B) Jβ single-chain germline region. An OR>1 represents a CD4+ bias, while an OR <1 represents a CD8+ bias with error bars representing the 95% confidence interval. The mean is represented by a red or black dot, with red representing statistical significance at the p < 0.05 by Fisher's exact test level after applying Bonferroni correction. (C) Significant (q < 0.05 by Fisher's exact test) log odds ratios reveals strong CD4+:CD8+ biases for 79 Jαβ pairs. (D) Boxplots were calculated for the set of all significant odds ratios associated with single chains (Jα or Jβ) and compared with those associated with Jαβ pairs. Paired associations for both CD4+ and (E) CD8+ status were significantly stronger (***p < 0.001 by Mann-Whitney U test) than those associated with a single chain alone. Associations for the J region were, overall, substantially weaker than those observed for the V chain. [file Image_2.JPEG]

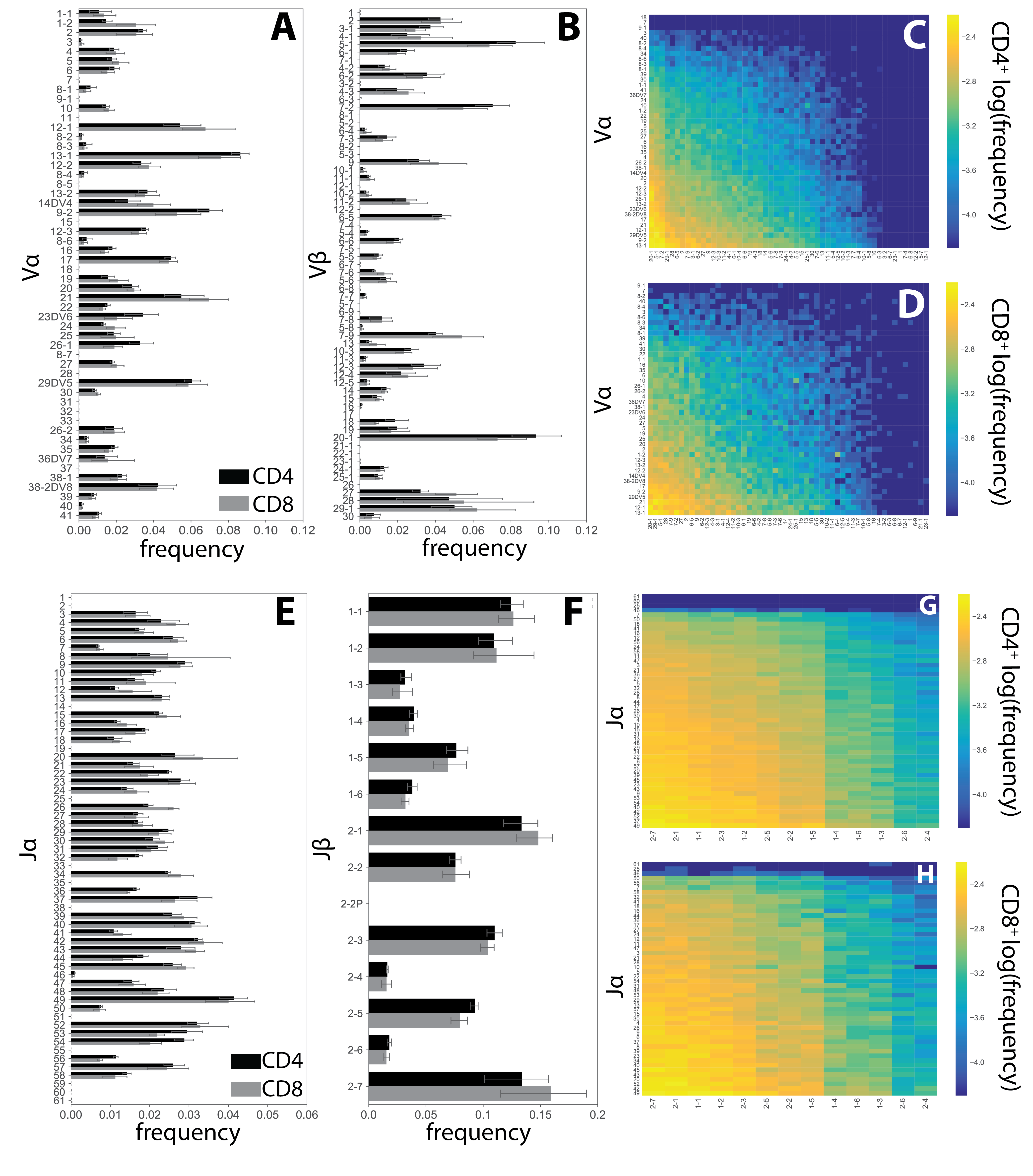

Supplement: Supplemental Figure 3 — V and J germline region usage. (A) Single-chain V region distributions for the α and (B) β chains. (C) Paired Vαβ usage for the CD4+ and (D) CD8+ T cell populations. (E) Single-chain J region distributions for the α and (F) β chains. (G) Paired Vαβ usage for the CD4+ and (H) CD8+ T cell populations. [file Image_3.JPEG]

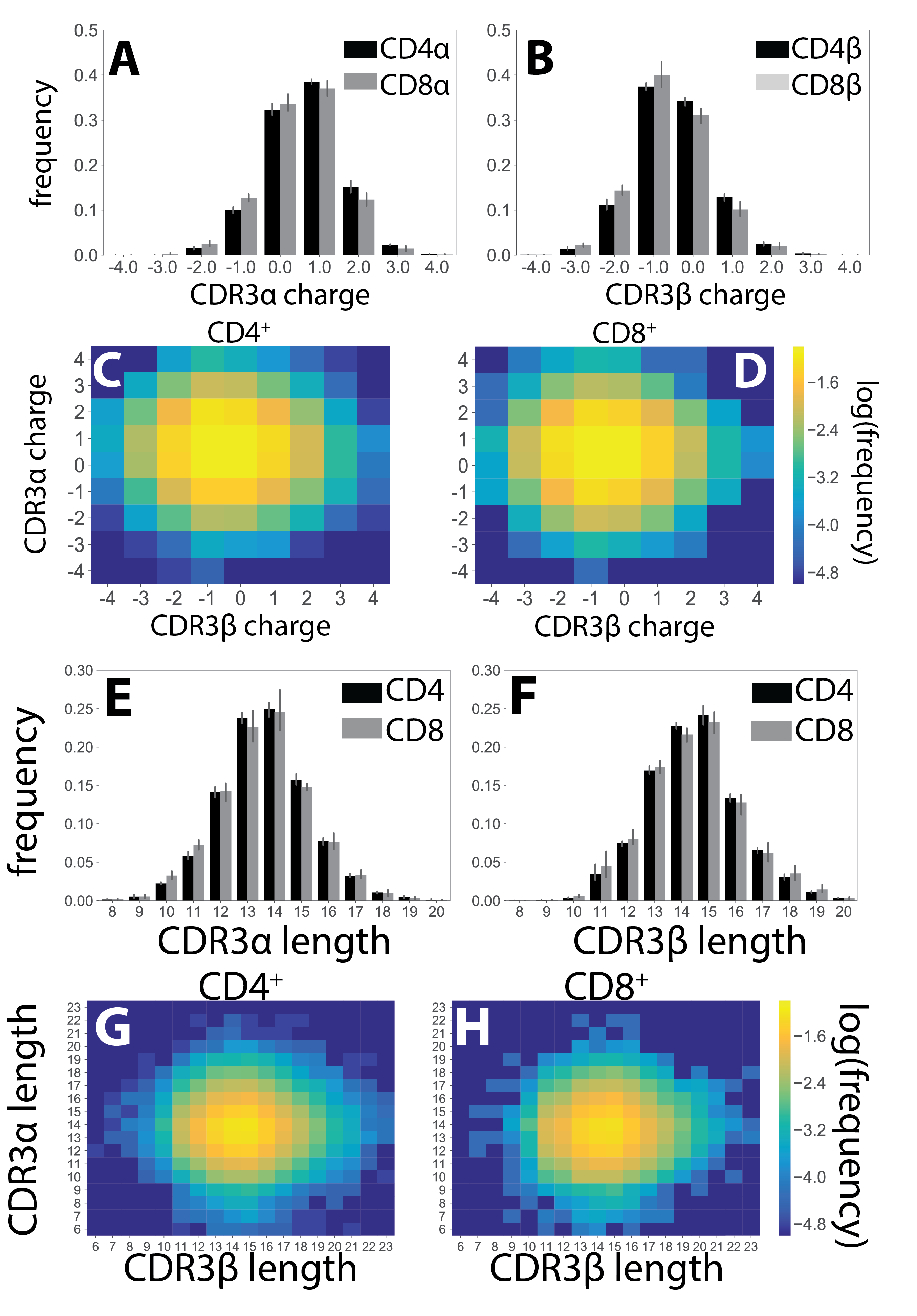

Supplement: Supplemental Figure 4 — CDR3 charge and length distributions. (A) Single-chain CDR3 charge for the α and (B) β chains, separated by CD4+ and CD8+ populations. (C) Paired CDR3αβ charge usage for the CD4+ and (D) CD8+ T cell populations. (E) Single-chain CDR3 length for the α and (F) β chains, separated by CD4+ and CD8+ populations. (G) Paired CDR3αβ length distributions for the CD4+ and (H) CD8+ T cell populations. [file Image_4.JPEG]

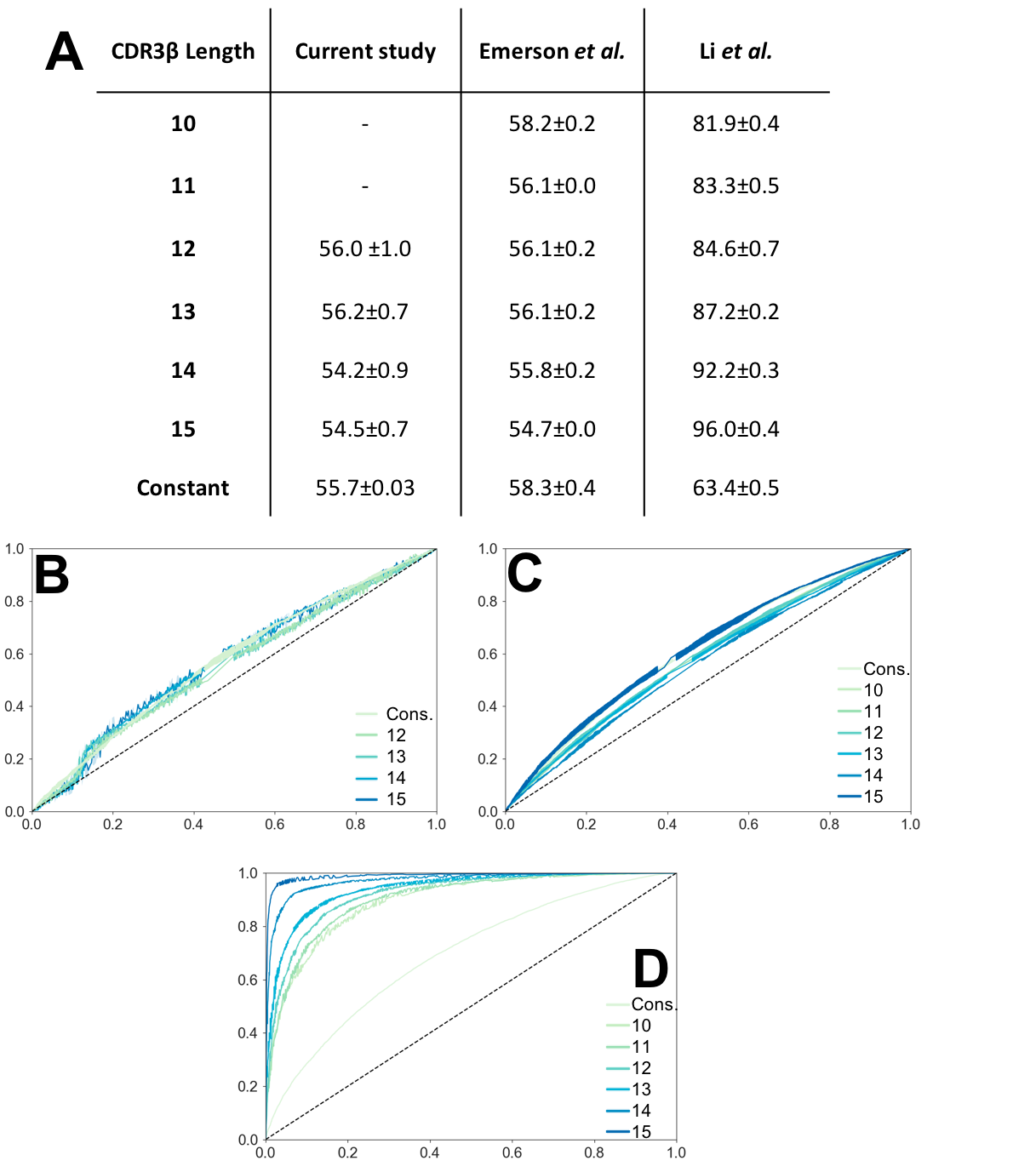

Supplement: Supplemental Figure 5 — SVM trained on CDR3β sequences converted to Atchley factors. A support vector machine (SVM) was trained on vectors composed of CDR3β sequences converted into numerical array according to their Atchley factors. As these vectors are dependent on the length of the CDR3 sequence, SVMs were trained separately for CDR3 sequences of lengths between 10 and 15, as previously done (23). For comparison, SVM accuracy for classifiers trained on CDR3β sequences converted to our constant length vector are also shown (Constant). (A) Accuracy for each model is reported as the percentage of correctly predicted CDR3 sequences using an independent testing set (25% of dataset). The Li et al. dataset is well-described by this SVM model, with accuracy as high as 96%. However, this model fails to accurately describe either the dataset used in this study or that of Emerson et al. (B) Receiver operator curves (ROC) for the current dataset, (C) the Emerson et al. dataset, and (D) the Li et al. dataset show length-dependent SVMs accurately predict the Li et al. dataset, but fail to do so for the other two datasets. [file Image_5.JPEG]

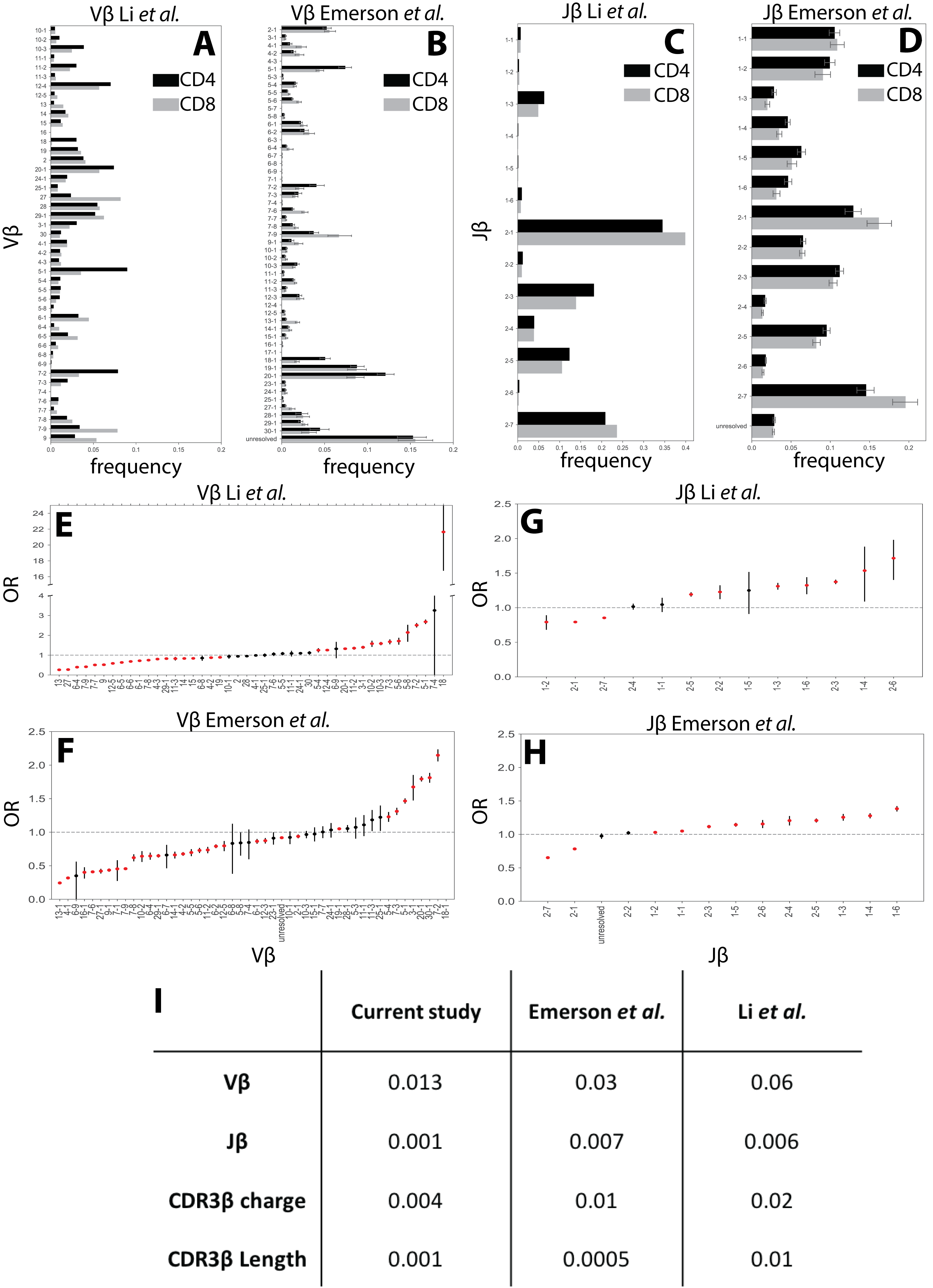

Supplement: Supplemental Figure 6 — V and J region usage patterns vary substantially between the Li et al. and Emerson et al. datasets. (A) β TCR sequences were obtained from 621,085 CD4+ and 64,725 CD8+ cells previously by Li et al. (23). Comparison of V-usage frequencies for each germline region reveals large differences between the CD4+ and CD8+ repertoires in this dataset. (B) V-usage frequencies observed by comparing 3,212,682 CD4+ and 1,774,260 CD8+ TCR sequences taken from Emerson et al. reveal less variation between the two cell types (24) and more closely resemble the results obtained in the present study (Supplemental Figure 3). (C) Similar results were obtained for Jβ region usage in the Li et al. and (D) Emerson et al. datasets. (E) We quantified the difference in V segment use in the CD4+ and CD8+ populations by calculating the odds ratio (OR) for each V region in the Li et al. dataset and (F) the Emerson et al. dataset independently. (G) Jβ usage between Li et al. dataset and (H) Emerson et al. datasets. (I) Mutual information with finite sampling correction was calculated for the association between β chain features (Vβ, Jβ, CDR3β length and charge) and lineage for the dataset used in this study (from Table), by Li et al. (23) and Emerson et al. (24). Substantially higher mutual information values, indicating stronger associations, were found for the Li et al. dataset as compared to the other two datasets. [file Image_6.JPEG]
