# Supplementary material for: Single T Cell Sequencing Demonstrates the Functional Role of αβ TCR Pairing in Cell Lineage and Antigen Specificity
Source: Front Immunol. 2019 Jul 31;10:1516. doi: 10.3389/fimmu.2019.01516 (PMC6684766; doi:10.3389/fimmu.2019.01516)
Supplement: Supplemental Table 1 — Demographic information for each subject. Peripheral blood mononuclear cells (PBMCs) were previously obtained from 5 healthy individuals (S1–S5) and sequenced using single-cell barcoding in emulsion (18, 19). The original PBMC samples from S1 and S3, as well as new samples from additional healthy individuals (S6, S7), were sequenced using a commercially available single-cell system (10× Genomics) (17). In all, we obtain 70,108 and 26,946 unique TCR pairs from CD4+ CD8+ T cells, respectively. Demographic information, as well as HLA types, are provided as available (19). [file Table_1.pdf]

Supplemental Table 1: **Demographic information for each subject.** Peripheral blood mononuclear cells (PBMCs) were previously obtained from 5 healthy individuals (S1-S5) and sequenced using single-cell barcoding in emulsion[18,19]. The original PBMC samples from S1 and S3, as well as new samples from additional healthy individuals (S6,S7), were sequenced using a commercially available single-cell system (10x Genomics)[17]. In all, we obtain 70,108 and 26,946 unique TCR pairs from $CD4^+$ $CD8^+$ T cells, respectively. Demographic information, as well as HLA types, are provided as available[19].

	Subject 1	Subject 2	Subject 3	Subject 4	Subject 5	Subject 6	Subject 7	Total
**Age (Gender)**	65 (Female)	69 (Male)	34 (Male)	33 (Female)	37 (Male)	-	-	-
**Platform(s)**	Abvitro + 10x	Abvitro	Abvitro + 10x	Abvitro	Abvitro	10x	10x	
**Unique CD4 pairs**	11,435+316	9,961	20,296+46	4,310	17,251	772	5,721	70,108
**Unique CD8 pairs**	4,649+27	2,007	2,652+33	7,049	6,658	101	3,770	26,946
**HLA-A**	24:02/24:02	03:01/26:01	68:01/68:02	02:01/02:01	-	-	-	
**HLA-B**	18:01/44:02	40:01/51:01	35:01/48:01	51:01/51:01	-	-	-	
**HLA-C**	05:01/07:01	01:02/03:04	04:01/08:01	01:02/14:02	-	-	-	
**HLA-DPA1**	01:03/01:03	01:03/02:01	01:03/01:03	01:03/01:03	-	-	-	
**HLA-DPB1**	04:02/20:01	04:01/14:01	04:01/04:02	04:01/04:01	-	-	-	
**HLA-DQA1**	01:01/05:05	01:01/03:02	01:02/03:01	01:02/03:03	-	-	-	
**HLA-DQB1**	03:01:05:01	03:03/05:01	03:02/05:02	03:02:06:02	-	-	-	
**HLA-DRB1**	01:01/11:01	01:01/05:01	04:04/16:01	04:01/15:01	-	-	-	
**HLA-DRB3/4/5**	3*02:02/Absent	3*01:01/Absent	4*01:03/5*02:02	01:03/01:01	-	-	-	
